# Promoting Mental Health Following the London Bombings: A Screen and Treat Approach

**DOI:** 10.1002/jts.20310

**Published:** 2008-02

**Authors:** Chris R Brewin, Peter Scragg, Mary Robertson, Monica Thompson, Patricia d'Ardenne, Anke Ehlers

**Affiliations:** Traumatic Stress ClinicLondon, UK; Institute of PsychotraumaLondon, UK; Centre for Anxiety Disorders and TraumaLondon, UK

## Abstract

Following the 2005 London bombings, a novel public health program was instituted to address the mental health needs of survivors. In this article, the authors describe the rationale for the program, characteristics of individuals assessed within the program, and preliminary outcome data. In addition to validated screening instruments and routine service usage data, standardized questionnaire outcome measures were collected. Seventy-one percent of individuals screened positive for a mental disorder. Of those receiving a more detailed clinical assessment, PTSD was the predominant diagnosis. Preliminary outcome data on 82 patients revealed large effect sizes for treatment comparable to those previously obtained in randomized controlled trials. The program succeeded in its aim of generating many more referrals of affected individuals than came through normal referral channels.

On July 7 and 21, 2005, two teams of four bombers attacked the London transport system. The July 7 attack was the largest mass casualty event in the United Kingdom since World War II, resulting in 775 casualties and 56 deaths from among the more than 4,000 passengers involved. On July 21, in contrast, the bombs failed to explode and no one was physically injured. Direct survivors of terrorist attacks have high rates of mental disorders (Whalley & Brewin, 2007), and based on previous experiences of disaster in the United States and the United Kingdom, a novel approach to managing the subsequent mental health needs was attempted. This involved the set up of a centralized screen and treat program to identify all affected individuals, screen them for mental disorders using validated measures, refer them for evidence-based treatment where appropriate, and monitor outcomes using standardized instruments. In this article, we describe how the approach differs from previous public health programs and provide interim data about the use of the service and clinical outcomes up to the end of December 2006.

In the United States, mental health programs initiated in response to disasters and terrorist attacks are funded by the Federal Emergency Management Agency (FEMA). The FEMA model, informed primarily by experience with natural disasters, assumes that large numbers of the population will be affected and that their responses likely indicate normal reactions to abnormal circumstances. It emphasizes crisis counseling and support services, along with outreach and public education for affected individuals. Consistent with this model, following the 1995 Oklahoma City bombing Project Heartland provided services to 9,345 individuals, most of whom were indirect community victims of the bombing (Call & Pfefferbaum, 1999). Subsequent evaluation has suggested that direct victims with more serious disorders may have been underserved in terms of screening, triage, referral to specialist services for established treatments, and subsequent monitoring. At the same time, mental health professionals may not have appreciated the need for outreach to detect individuals with established disorders (Call & Pfefferbaum, 1999).

Similar issues were identified in the FEMA-funded Project Liberty that was instituted following the September 11, 2001, attacks and that provided crisis counseling to over 690,000 individuals. A survey conducted 6 months after the attacks concluded that there was substantial unmet need for treatment for posttrau- matic stress disorder (PTSD) or depression, and that this was particularly marked in individuals with no previous contact with mental health services (Stuber et al., 2006). After approximately 2 years, an enhanced services program was approved for individuals with severe and persistent symptoms (Donahue, Lanzara, Felton, Essock, & Carpinello, 2006). This consisted of a screening instrument applied to those already receiving crisis counseling, longer evidence-based brief counseling interventions, and training and technical assistance for selected clinicians. Training was provided in a specially developed 10–12 session cognitive–behavioral intervention. Interviews conducted 7 weeks apart with recipients of enhanced services found a reduction in depression and grief symptoms and some aspects of impairment, but no significant fall in PTSD symptoms (Donahue, Jackson, Shear, Felton, & Essock, 2006).

In the United Kingdom, the need for a longer-term mental health response was identified after a number of disasters including the King's Cross fire in 1987. Proposals were made after this incident for a response that included immediate support and counseling coupled with the identification of all trauma-exposed persons, to be followed by a formal outreach program involving screening and treatment (Turner, Thompson, & Rosser, 1989). This early work was supplemented by a growing appreciation that most psychological responses to trauma are short-term and resolve naturally, as well as by concern about the effectiveness of mass interventions such as psychological debriefing for trauma-exposed individuals (Rose & Bisson, 1998).

A similar screen and treat approach has been proposed that also distinguishes between immediate and longer-term intervention (Brewin, 2001). Immediate intervention was described as “psychological first aid” over 50 years ago (Drayer et al., 1954), and the principles have recently been updated (Young, 2006). The novel emphasis in the screen and treat program is primarily on the longer-term goal of identifying, following up, and screening all trauma-exposed individuals with properly validated measures to determine who develops persistent symptoms of psychopathol- ogy, and then giving them evidence-based treatment. A similar emphasis on outreach was an important aspect of the mental health response to the 1998 bombing in Omagh, Northern Ireland (Gillespie et al., 2002). The importance of outreach is underscored by reports that there is a particularly low rate of treatment-seeking for PTSD, with fewer than 10% of affected individuals receiving any kind of help in the year after onset (Wang et al., 2005).

## METHOD

### The London Bombings Trauma Response Programme

Fifteen days after the July 7 bombings, a Psychosocial Steering Group convened by the London Development Centre for Mental Health (part of the National Care Services Improvement Partnership), met to coordinate the mental health response, with representation from mental health agencies, specialist trauma centers, health commissioners, primary care physicians, emergency services, first-response agencies, and survivor groups. The group considered, approved, and sought funding from the U.K. Department of Health for two response elements, a central screening team and additional treatment resources. The treatment resources represented an extension of existing services offered by qualified clinical psychologists working and being supervised within specialist, multidisciplinary psychological trauma centers in London. All therapy was free of charge and travel costs were reimbursed where appropriate, in line with standard procedures. The primary focus of treatment was expected to be posttraumatic stress disorder (PTSD), and consistent with the recently published official guidelines for the management of PTSD (National Institute for Clinical Excellence, 2005), two treatments were considered acceptable (trauma-focused cognitive—behavior therapy [CBT] and eye movement desensitization and reprocessing [EMDR]). All lead clinicians of the participating treatment centers met monthly to ensure treatments were provided with uniform quality and in strict adherence to NICE guidelines.

Trauma-focused CBT comprises a group of treatment programs that have in common that they involve imaginal and in vivo exposure to the memory and reminders of the bombings coupled with cognitive therapy. Rather than working from specific treatment manuals, clinicians were required to implement the individual trauma-focused CBT or EMDR programs used in their respective trauma specialist center and received ongoing supervision from experienced trauma clinicians within their center. The most commonly used approach was trauma-focused CBT (>80%). A minority of patients received either a combination of CBT and EMDR (≈ 10%), or EMDR only (< 10%). There were no restrictions on number of sessions. Evidence-based CBT was also used for other disorders where necessary.

The most innovative part of the program was the establishment of a dedicated screening team charged with collating information about individuals involved in the bombings and identifying those with bombing-related mental disorders, as well as providing advice to professionals and the public on demand. The service was widely advertised to health professionals and in the national and London print and broadcast media. A dedicated helpline hosted by NHS Direct, a 24-hour telephone-based consultation service that provides medical advice to the general population, was also set up with the aim of referring appropriate callers to the screening team. In addition to self-referrals and referrals from medical practitioners, the service was advertised to users of the 7 July Assistance Centre, a Government-funded center set up under emergency planning legislation to respond to immediate needs for information, advice, and counseling. Lists of names of survivors were also provided by hospitals that had treated them, by the London Bombings Charitable Relief Fund, and by the Health Protection Agency.

All individuals identified in this way received a letter or telephone call and a brief 2-page questionnaire establishing their contact details, basic demographic facts including the age and gender of any children living with them, the extent of their involvement in the bombings, and screening questions to detect any current symptoms of psychopathology. If they had any children living with them, they were sent additional materials so the children could be screened for symptoms as well. Information about the screening team was also widely disseminated via the Metropolitan Police witness list, and via the occupational health departments serving members of the emergency services attending the incident. Affected members of the emergency services could opt to be seen within their own organization or to attend the Trauma Response Programme.

### Measures

The Trauma Screening Questionnaire (TSQ: Brewin et al., 2002) was used to screen for the presence of posttraumatic stress disorder. The TSQ is a 10-item measure with a yes/no response scale enquiring about the presence of each symptom at least twice in the past week. Previous research has demonstrated that it has excellent performance relative to other instruments and that endorsement of six or more symptoms yields high levels of sensitivity and specificity (Brewin, 2005; Walters, Bisson, & Shepherd, 2007). This was supplemented by a 2-item depression screener that anchored previously validated items (Kroenke, Spitzer, & Williams, 2003) to observed changes since the bombings, and by a 1-item travel phobia screener ["Since the bombings, has your daily life become difficult because you felt unable to use public transport (e.g., not being able to get to work, to get your shopping done, or to get to social events) or because you felt very distressed when using public transport?”]. These were supplemented by three more general items designed to detect alternative ways of expressing distress, “Since the bombings have you noticed that you have been smoking much more?”; “Since the bombings have you noticed that you have been drinking much more alcohol?”; “Since the bombings have you noticed any other reaction that is a concern to you?” For consistency, all items were answered on a yes/no response scale. Very few children were involved in the London bombings and the relevant data are therefore not included in this report.

### Procedure

The screening team commenced operation in September 2005, sending out information about trauma responses and the two-page brief questionnaire. Individuals screening positive on the TSQ or endorsing any of the additional screening items were invited for a more detailed assessment that included the Structured Clinical Interview for DSM-IV (SCID: First et al., 1997), the CAGE alcohol abuse screening instrument (Mayfield, McLeod, & Hall, 1974), the SF-12 Health Survey (Ware, Kosinski, & Keller, 1996) and, where appropriate, the Short McGill Pain Questionnaire (Melzack, 1987) and the Inventory of Complicated Grief-Revised (Prigerson & Jacobs, 2001).

This longer clinical assessment had a number of aims. The first was to identify individuals with preexisting mental health problems, such as psychosis or substance abuse, and to either refer them back to their treating clinicians or arrange for appropriate treatment for these problems. The second was to determine suitability for trauma-focused treatment. Suitability was primarily defined in terms of meeting the *Diagnostic and Statistical Manual of Mental Disorders, Fourth Edition (DSM-IV*; American Psychiatric Association, 1994) criteria or *International Classification of Diseases (ICD-10;* World Health Organization, 1992) criteria for a disorder that was related to being exposed to the bombings and that was not resolving of its own accord. Conditions not meeting full diagnostic criteria were also eligible if they were persistent and were associated with significant distress or impairment. Based on the self-reported trajectory of symptoms, a clinical decision was made whether to refer for immediate treatment or to continue monitoring in the expectation that recovery would occur naturally. In the latter case, individuals were followed up at 3-, 6-, and 9-month intervals to determine that symptoms had indeed resolved satisfactorily. With their permission, repeated screening was routinely employed with all individuals contacting the screening team, regardless of their diagnostic status, to guard against delayed-onset PTSD and PTSD that gradually worsened over time or was exacerbated by subsequent events. A recent review has indicated that approximately 15% of cases of civilian PTSD fall into this category (Andrews, Brewin, Philpott, & Stewart, 2007).

## RESULTS

By the end of May 2007, 14 bombing survivors had been referred for treatment by their family doctors to the participating treatment centers using standard referral procedures in place before the Trauma Response Programme, and a further 19 referrals to other nonparticipating centers or private psychologists had been recorded. In the same period, the Trauma Response Programme identified 906 named individuals of whom to date 596 individuals have completed the initial screening with the two-page questionnaire. Of these 596 individuals, 370 were invited for a detailed assessment, of whom 24 (6%) did not attend. Of the 346 receiving detailed assessment, 91 (26%) were judged as requiring monitoring only, whereas 255 (74%) were referred for treatment. Among the 255 referred by the screening team, their primary diagnoses were: 184 (72%) *DSM-IV* or *ICD-10* PTSD (with or without comorbid disorders), 20 (8%) travel phobia, 22 (9%) adjustment disorder, 10 (4%) complicated grief, 7 (3%) generalized anxiety disorder, 5 (2%) major depressive disorder, and 7 (3%) other diagnoses.

Preliminary outcome data are available on 60 individuals with *DSM-IV* PTSD and 22 individuals with *ICD-10* PTSD who were referred to the specialist trauma centers. There were 28 men and 54 women with an average age of 35.2 years (range 19–57 years). Of this group, six never attended, one was referred on to another service, and 75 started treatment; 72 completed treatment and 3 dropped out after two sessions each. All patients receiving treatment within the program, including two of the dropouts, completed the Posttraumatic Diagnostic Scale (PDS: Foa et al., 1997) and the Beck Depression Inventory (BDI: Beck, Ward, Mendelson, Mock, & Erbaugh, 1961) at each session. This was done to ensure completeness of data in case patients did not attend the last treatment sessions. Thus, the outcome data are intent-to-treat.

Because the diagnostic criteria for PTSD in the *DSM-IV* are more stringent than in *ICD-10,* the analyses distinguished between people who would meet full *DSM-IV* criteria and those would meet *ICD-10,* but not *DSM-IV* criteria. At the first treatment session, those with *DSM-IV* PTSD had a mean PDS score of 33.76 (*SD* = 9.37) and a mean BDI score of 25.16 (*SD* = 9.30), whereas those with *ICD-10* PTSD had a PDS score of 21.62 (*SD* = 10.01) and a BDI score of 14.09 (*SD* = 10.59). The modal number of treatment sessions was nine (range = 1–29).

At the final treatment session, those with *DSM-IV* PTSD had a mean PDS score of 9.58 (*SD* = 9.78) and a mean BDI score of 8.72 (*SD* = 8.03), whereas those with *ICD-10* PTSD had a PDS score of 6.10 (*SD* = 5.57) and a BDI score of 5.00 (*SD* = 6.96). A mixed model ANOVA on the PDS scores with group *(DSM-IV* vs. *ICD-10)* as a between-subjects factor and time (first vs. last treatment session) as a within-subjects factor yielded significant effects of group, *F* (1, 72) = 18.40, *p* < .001, and time, *F* (1, 72) = 161.41, *p* < .001, and a significant Group x Time interaction, *F*(1, 72) = 7.45, *p* < .01. The pretreatment and posttreatment effect size *d* (calculated as the difference between the pretreatment and posttreatment means divided by their common standard deviation) was 2.53 for *DSM-IV* PTSD and 1.99 for *ICD-10* PTSD. Following Jacobson and Truax (1991), clinically significant change was defined as a posttherapy PDS score closer to the mean of a functional trauma-exposed population than to the mean of a population with PTSD. Mean PDS scores for functional and PTSD populations were taken from Foa et al. (1997), yielding a cutoff value of 24. In Foa et al.'s study, the mean PDS score for the PTSD sample was almost identical to our group with *DSM-IV* PTSD. Forty-six out of 53 (87%) of our *DSM-IV* PTSD cases for whom data were available showed clinically significant change.

A similar mixed model ANOVA on the BDI scores yielded significant effects of Group, *F*(1, 72) = 14.78, *p* < .001, and Time, *F*(1, 72) = 102.39, *p* < .001, and a significant Group × Time interaction, *F*(1, 72) = 8.12, *p* < .01. The pretreatment and post-treatment effect size was 1.90 for *DSM-IV* PTSD and 1.04 for *ICD-10* PTSD. Mean BDI scores for functional and depressed populations were taken from Seggar, Lambert, and Hansen (2002), who suggest that they can be discriminated by a cutoff value of 15. In their study, the mean BDI score for the depressed sample was almost identical to our group with *DSM-IV* PTSD. Forty-two out of 53 (79%) of our *DSM-IV* PTSD cases for whom data were available showed clinically significant change at posttreatment.

## DISCUSSION

This was the first time that the public health response to a major terrorist attack has been based around a team dedicated to identifying and assessing survivors, screening using validated screening instruments, evidence-based interventions, and session-by-session outcome assessment using standardized measures. In contrast to many previous programs, a very limited emphasis was put on crisis counseling (although this was available on demand from the 7 July Assistance Centre). Outreach efforts were specifically focused on screening and advising directly affected individuals rather than on public education and counseling more generally. Data from usage of the screening team indicate that 31.9% of those referred had a bombings-related mental disorder of some kind, and that in over 72% of these the primary problem was PTSD. These figures are consistent with estimates that between 30% and 40% of those directly exposed to a terrorist attack are likely to develop PTSD (Whalley & Brewin, 2007).

During the first 15 months of the Trauma Response Programme, the screening team identified 255 bombing survivors with mental health problems severe enough to require treatment. In contrast, only 14 survivors were referred for treatment to the same participating treatment centers by their family doctors during this period. Although all family doctors in London had been alerted to the increased risk of mental health problems including PTSD in two letters soon after the bombings, very few patients were identified through routine medical care. This discrepancy underscores the role of outreach programs in providing rapid treatment of mental health problems after terrorist attacks.

The preliminary outcome data add to previous evidence (Gillespie et al., 2002) that established psychological treatment methods can substantially reduce PTSD following terrorist attacks. Reassuringly, in view of uncertainty about the impact on PTSD symptoms of the enhanced counseling delivered within Project Liberty (Donahue, Jackson et al., 2006), the effect sizes obtained were comparable to those previously achieved in randomized controlled trials (Clark, 2004) and in the Omagh audit (Gillespie et al., 2002). Follow-up data are currently being collected to confirm that these represent lasting rather than temporary effects of treatment.

At the end of treatment, the mean scores on the PDS were below the clinical range of symptoms, suggesting that the majority of patients recovered. The absence of a waiting list control group, however, makes it difficult to establish with certainty that the good outcome was due to treatment rather than natural recovery. However, a previous randomized controlled trial that used an outreach and screening approach in motor accident vehicle survivors showed that trauma-focused CBT is superior to a waiting-list control and to self-help (Ehlers et al., 2003). The treatment effect size of *d* = 2.5 for the PDS observed in the Trauma Response Programme was equivalent to that reported in this trial, and much larger than the *d* = .70 observed for the wait-list condition. As there is no reason to believe that bombing survivors would have greater natural recovery than accident survivors, we can assume that the treatment led to greater improvement than would be expected on the basis of natural recovery alone.

Evaluation of this and similar trauma response programs following disasters and terrorist attacks pose a number of important methodological challenges. For ethical and logistical reasons, random assignment to no-treatment or waiting-list control groups will rarely be acceptable. Nevertheless, it is important to evaluate whether any interventions actually improve survivors' mental health, as reviews have indicated that some post-trauma interventions may actually worsen symptoms such as PTSD (McNally, Bryant, & Ehlers, 2003). Ensuring adherence to standardized treatment protocols is likely to come second to managing the surge in demand for treatment and the sudden need to recruit trauma clinicians. Nevertheless, as resources are limited, the most promising approach appears to offer a range of evidence-based treatments rather than treatments of unproven efficacy, and to evaluate them under real-world constraints. The outcomes and cost-effectiveness of the Trauma Response Programme are undergoing more lengthy and formal evaluation. We nevertheless believe it is of value to share our experiences at this stage to inform global concerns about how to plan for the consequences of disasters and terrorism.

## References

[b1] American Psychiatric Association (1994). Diagnostic and statistical manual of mental disorders.

[b2] Andrews B, Brewin CR, Philpott R, Stewart L (2007). Delayed onset post-traumatic stress disorder: A systematic review of the evidence. American Journal of Psychiatry.

[b3] Beck AT, Ward CH, Mendelson M, Mock J, Erbaugh J (1961). An inventory for measuring depression. Archives of General Psychiatry.

[b4] Brewin CR (2001). Cognitive and emotional reactions to traumatic events: Implications for short-term intervention. Advances in Mind-Body Medicine.

[b5] Brewin CR (2005). Systematic review of screening instruments for the detection of posttraumatic stress disorder in adults. Journal of Traumatic Stress.

[b6] Brewin CR, Rose S, Andrews B, Green J, Tata P, McEvedy C (2002). A brief screening instrument for posttraumatic stress disorder. British Journal of Psychiatry.

[b7] Call JA, Pfefferbaum B (1999). Lessons from the first two years of Project Heartland, Oklahoma's mental health response to the 1995 bombing. Psychiatric Services.

[b8] Clark DM (2004). Developing new treatments: On the interplay between theories, experimental science and clinical innovation. Behaviour Research and Therapy.

[b9] Donahue SA, Jackson CT, Shear KM, Felton CJ, Essock SM (2006). Outcomes of enhanced counseling services provided to adults through Project Liberty. Psychiatric Services.

[b10] Donahue SA, Lanzara CB, Felton CJ, Essock SM, Carpinello S (2006). Project Liberty: New York's crisis counseling program created in the aftermath of September 11, 2001. Psychiatric Services.

[b11] Drayer CS, Cameron DC, Woodward WD, Glass AJ (1954). Psychological first aid in community disasters—prepared by the American Psychiatric Association Committee on Civil Defense. Journal of the American Medical Association.

[b12] Ehlers A, Clark DM, Hackmann A, McManus F, Fennell M, Herbert C (2003). A randomized controlled trial of cognitive therapy, self-help booklet, and repeated assessment as early interventions for PTSD. Archives of General Psychiatry.

[b13] First MB, Spitzer RL, Gibbon M, Williams JBW (1997). Structured Clinical Interview for DSM-IV Axis I Disorders—Patient Edition (SCID-IP; Version 2.0, 4. 97 revision).

[b14] Foa EB, Cashman L, Jaycox L, Perry K (1997). The validation of a self-report measure of posttraumatic stress disorder: The Posttraumatic Diagnostic Scale. Psychological Assessment.

[b15] Gillespie K, Duffy M, Hackmann A, Clark DM (2002). Community based cognitive therapy in the treatment of posttraumatic stress disorder following the Omagh bomb. Behaviour Research and Therapy.

[b16] Jacobson NS, Truax P (1991). Clinical significance: A statistical approach to defining meaningful change in psychotherapy research. Journal of Consulting and Clinical Psychology.

[b17] Kroenke K, Spitzer RL, Williams JBW (2003). The Patient Health Questionnaire-2: Validity of a two-item depression screener. Medical Care.

[b18] Mayfield D, McLeod G, Hall P (1974). The CAGE questionnaire: Validation of a new alcoholism instrument. American Journal of Psychiatry.

[b19] McNally RJ, Bryant RA, Ehlers A (2003). Does early psychological intervention promote recovery from posttraumatic stress?. Psychological Science in the Public Interest.

[b20] Melzack R (1987). The short-form McGill Pain Questionnaire. Pain.

[b21] National Institute for Clinical Excellence (2005). Post-traumatic stress disorder: The management of PTSD in adults and children in primary and secondary care.

[b22] Pfefferbaum B, North CS, Flynn BW, Norris FH, DeMartino R (2002). Disaster mental health services following the 1995 Oklahoma City bombing: Modifying approaches to address terrorism. CNS Spectrums.

[b23] Prigerson HG, Jacobs SC, Stroebe MS, Hansson RO, Stroebe W, Schut H (2001). Traumatic grief as a distinct disorder: A rationale, consensus criteria, and a preliminary empirical test. Handbook of bereavement research: Consequences, coping, and care.

[b24] Rose S, Bisson JI (1998). Brief early psychological interventions following trauma: A review of the literature. Journal of Traumatic Stress.

[b25] Seggar LB, Lambert MJ, Hansen NB (2002). Assessing clinical significance: Application to the Beck Depression Inventory. Behavior Therapy.

[b26] Stuber J, Galea S, Boscarino JA, Schlesinger M (2006). Was there unmet mental health need after the September 11, 2001 terrorist attacks?. Social Psychiatry and Psychiatric Epidemiology.

[b27] Turner SW, Thompson JA, Rosser RM (1989). The King's Cross fire: Planning a “phase two” psychosocial response. Disaster Management.

[b28] Walters JTR, Bisson JI, Shepherd JP (2007). Predicting post-traumatic stress disorder: Validation of the Trauma Screening Questionnaire in victims of assault. Psychological Medicine.

[b29] Wang PS, Berglund P, Olfson M, Pincus HA, Wells KB, Kessler RC (2005). Failure and delay in initial treatment contact after first onset of mental disorders in the National Comorbidity Survey replication. Archives of General Psychiatry.

[b30] Ware J, Kosinski M, Keller SD (1996). A 12-Item Short-Form Health Survey: Construction of scales and preliminary tests of reliability and validity. Medical Care.

[b31] Whalley MG, Brewin CR (2007). Mental health following terrorist attacks. British Journal of Psychiatry.

[b32] World Health Organization (1992). International classification of diseases.

[b33] Young BH, Ritchie EC, Friedman MJ, Watson PJ (2006). The immediate response to disaster: Guidelines to adult psychological first aid. Mass violence and early intervention.

